# Supplementary material for: The ATG8 Family Proteins GABARAP and GABARAPL1 Target Antigen to Dendritic Cells to Prime CD4+ and CD8+ T Cells
Source: Cells. 2022 Sep 6;11(18):2782. doi: 10.3390/cells11182782 (PMC9496894; doi:10.3390/cells11182782)
Supplement: Supplementary file 1 [file cells-11-02782-s001.zip › cells-1771965-supplementary.pdf]

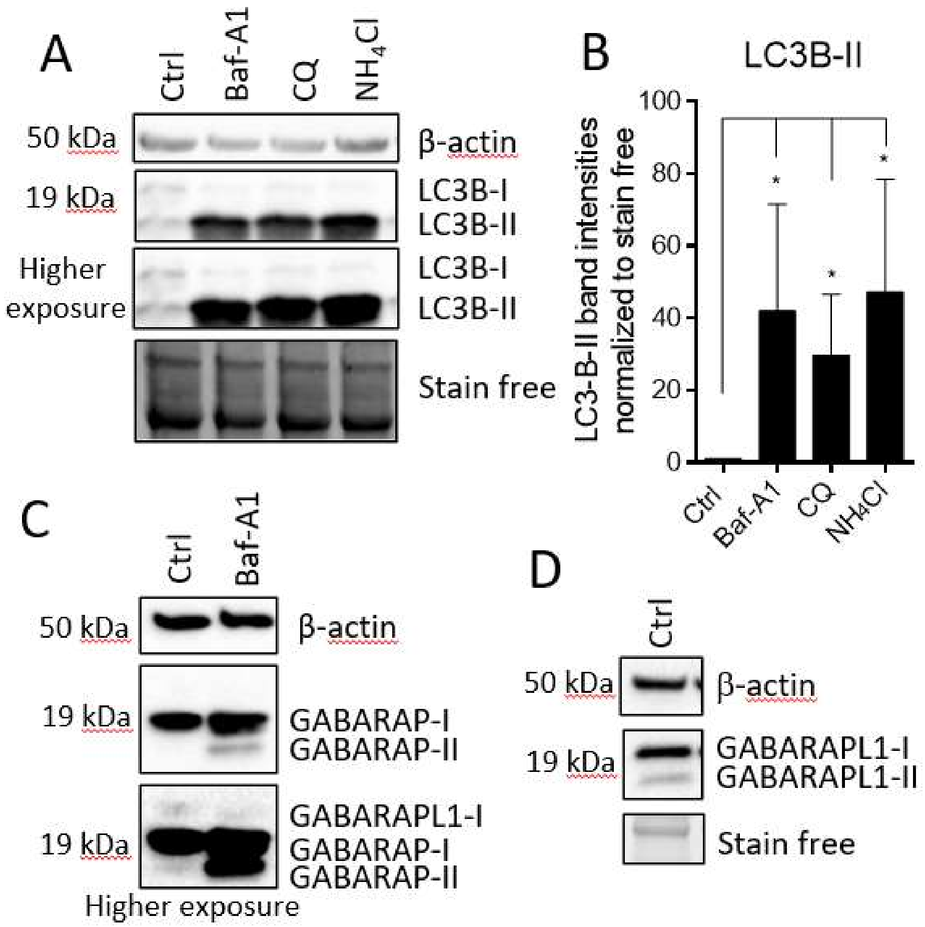

Figure S1 : dendritic cells have basal autophagic flux. BmDC expressed GABARAP and GABARAPL1 proteins. BmDC were treated during 2 h with different autophagic inhibitors: chloroquine (CQ, 40 µM); bafilomycine-A1 (Baf-A1, 100 nM) and ammonium chloride (NH_4Cl, 50 mM) at day 6 of differentiation. A) Cells lysates were analyzed by western blotting using anti-LC3B and anti-ACTIN antibodies on 12 % TGX gel. B) Autophagic flux was quantified by western blotting analysis of LC3B-II protein band intensities. LC3B-II band intensities were normalized to stain free band using Biorad stain free technologies. Statistic test: unpaired t-test, *: $P<0,05$. The histogram was representative of 4 independent experiments. Error bars represent ± SD. C) The expression of GABARAP and GABARAPL1 proteins were analyzed by western blotting on 12 % TGX gel using an anti-GABARAP-

GABARAPL1 antibodies. D) The expression of GABARAPL1 proteins was also analyzed using an anti-GABARAPL1 antibodies by western blotting.

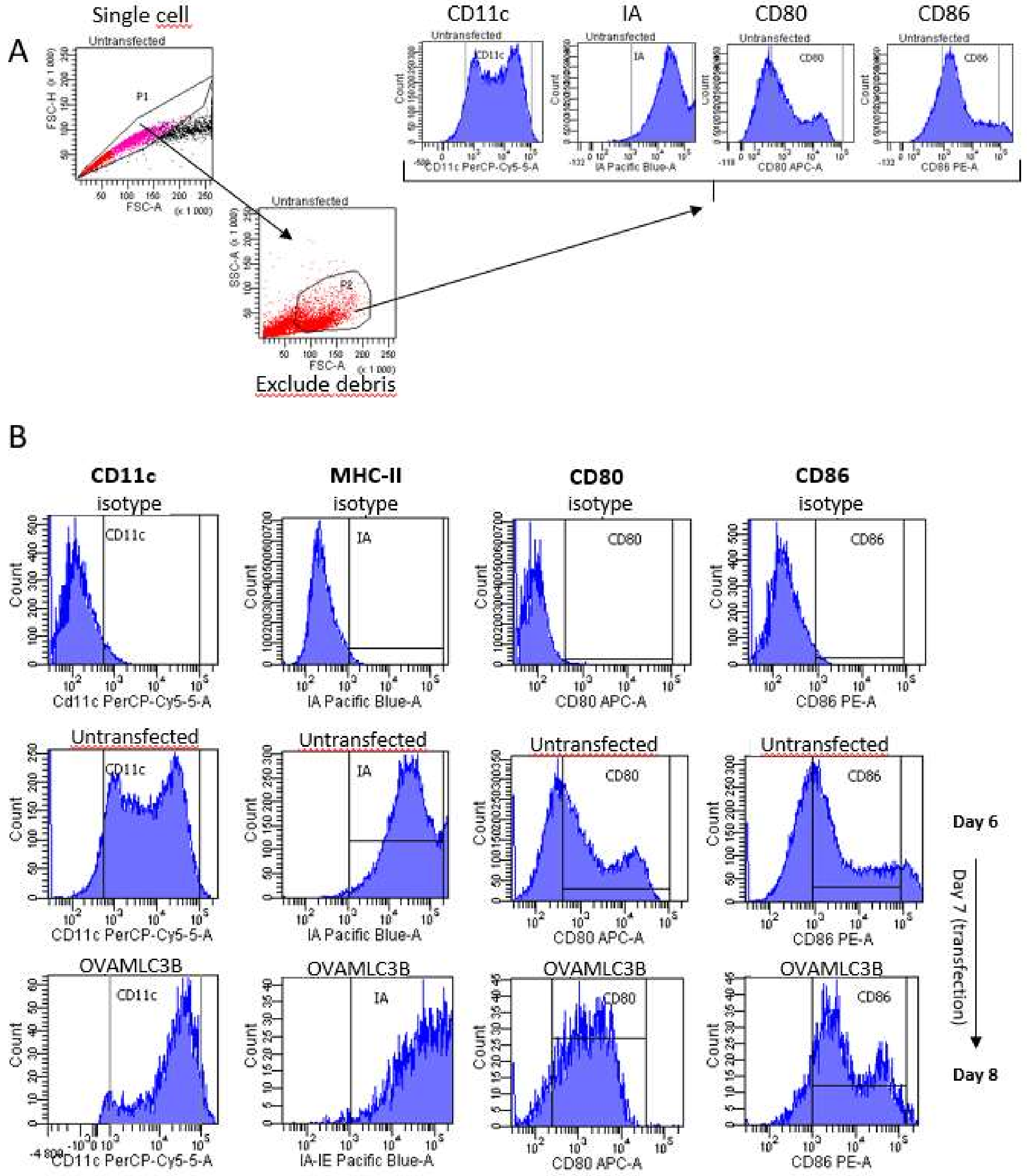

Figure S2: Expression of CD11c, CMH-II, CD80, and CD86 on bmDCs were detected by flow cytometry prior (D6) and after (D8) transfection. A) Flow cytometry strategy gating for the expression of CD11c, IA, and costimulatory molecules CD80 and CD86 after 6 days of bone marrow cells differentiation after 6 days. B) BmDC dendritic cells were transfected by the JETPEI-Macrophage kit plasmid vector encoding the OMLC3B protein at D7.
